# Gestational Hypertension as Risk Factor of Hypertension in Middle-Aged and Older Women

**DOI:** 10.3390/ijerph17114052

**Published:** 2020-06-05

**Authors:** Mariko Watanabe, Toshimi Sairenchi, Keiko Nishida, Koji Uchiyama, Yasuo Haruyama, Hiroshi Satonaka, Toshihiko Ishimitsu, Takanori Yasu, Ichio Fukasawa, Gen Kobashi

**Affiliations:** 1 Department of Obstetrics and Gynecology, Dokkyo Medical University, Mibu, Tochigi 321-0293, Japan; i-fuka@dokkyomed.ac.jp; 2 Department of Public Health, School of Medicine, Dokkyo Medical University, Mibu, Tochigi 321-0293, Japan; tossair@dokkyomed.ac.jp (T.S.); keikonsd@dokkyomed.ac.jp (K.N.); yasuo-h@dokkyomed.ac.jp (Y.H.); genkoba@dokkyomed.ac.jp (G.K.); 3 Department of Obstetrics and Gynecology, Faculty of Medicine, University of Tsukuba, Tsukuba, Ibaraki 305-8575, Japan; 4 Laboratory of International Environmental Health, Center for International Cooperation, Dokkyo Medical University, Mibu, Tochigi 321-0293, Japan; koji-u@dokkyomed.ac.jp; 5 Department of Nephrology and Hypertension, Dokkyo Medical University, Mibu, Tochigi 321-0293, Japan; satonaka@dokkyomed.ac.jp (H.S.); isimitu@dokkyomed.ac.jp (T.I.); 6 Department of Cardiovascular Medicine and Nephrology, Dokkyo Medical University Nikko Medical Center, Nikko, Tochigi 321-2593, Japan; tyasu@dokkyomed.ac.jp

**Keywords:** gestational hypertension, hypertensive disorders of pregnancy, hypertension, risk factor, cardiovascular disease

## Abstract

The association of gestational hypertension (GH) with future hypertension in Japanese women is unclear. Hence, this study aimed to examine the association between GH and the risk of future hypertension in middle-aged-to-older Japanese women. A case-control study was performed, including 62 hypertensive women (case) and 75 nonhypertensive women (control). GH during the first pregnancy was diagnosed on the basis of the Maternal and Child Health Handbook record. Hypertensive women were recruited from outpatients in the hospital and residents who completed an annual health check-up in a community. Hypertension was defined as blood pressure with systolic blood pressure ≥140 mmHg and/or diastolic blood pressure ≥90 mmHg, or taking antihypertensive medications. The average age (SD) of the cases and controls at the time of recruitment was 63.1 (8.4) and 57.7 (9.4), respectively. The multivariable-adjusted odds ratio of GH for hypertension in middle-aged-to-older women was 4.2 (95% confidence interval, 1.0–17.5) after adjustment for potential confounding factors such as age and body-mass index (BMI) upon recruitment, prepregnancy BMI, and age at first delivery. In conclusion, GH can be an independent risk factor for future hypertension among Japanese women.

## 1. Introduction

Hypertension is globally a well-known risk factor for cardiovascular diseases. Hypertension in middle-aged women is becoming increasingly important because of the longer life expectancy in women. While men were the focus of studies in the past decades, hypertension and cardiovascular diseases for women are now being investigated [1]. Treatments in the early stages of hypertension, prevention of hypertension in women by identifying high-risk populations, and continuous intervention are the most important identified issues.

Recently, several types of hypertensive disorders in pregnancy (HDP) were suggested as risk factors for future hypertension according to studies in many countries [2,3,4,5,6,7,8]. HDP, which occurs in 5–10% of all pregnancies, remains one of the most common causes of maternal and/or fetal death [9,10]. HDP was believed to have several pathogeneses on the basis of the complex interaction of genetic and environmental factors [11,12,13,14,15,16,17]. HDP is divided into four clinical types [9]. In women without hypertension before pregnancy, (1) pre-eclampsia (PE) is defined as the incidence of hypertension with proteinuria during pregnancy (blood pressure ≥140/90 mmHg and proteinuria ≥300 mg/day after the 20th week of gestation), with the normalization of blood pressure and proteinuria after delivery; and (2) gestational hypertension (GH) is defined as the incidence of hypertension during pregnancy, with the normalization of blood pressure after delivery (without the occurrence of PE). In women with hypertension or proteinuria before pregnancy, (3) superimposed pre-eclampsia (SPE) is defined as the incidence of proteinuria and/or hypertension or progression to severe proteinuria, and/or hypertension during pregnancy; and (4) chronic hypertension (CH) is defined as hypertension before pregnancy (except SPE). Women with SPE or CH are expected to be monitored continuously for hypertension or proteinuria after pregnancy by their family doctor or internal physician. Meanwhile, women who have either of the two former types, especially GH, do not require special care because their blood pressure returned to normal after delivery.

Previous studies suggested an association between GH and/or PE with future hypertension [2,3,4,5,6,7,8]. In these studies, meta-analyses reported the association of PE with the risk of future hypertension in Western and Asian countries [2,3], and several epidemiological studies reported the association of GH with the risk of future hypertension in non-Asian populations [4,5,6,7,8]. In Japan, a few studies discussed the association between GH and/or PE and future hypertension [18,19]. A study reported that middle-aged-to-older women with HDP who were not divided into subtypes such as GH were more likely than women without HDP to use antihypertensive medications (2.9% vs. 13.9%; odds ratio (OR) 4.28, 95% confidence interval (CI) 2.14–8.57) [18]. Another study showed an association between a 10 mmHg increase in diastolic blood pressure during pregnancy, which was not a GH definition, and a 1.70-fold increase in the risk of hypertension in middle-aged-to-older women [19]. These studies had an unclear definition of hypertension or did not distinguish between GH and PE. Lack of evidence exists of the association of GH with future hypertension for Asian countries. Studies of the association of GH and/or PE with future hypertension in Japanese women are necessary because evidence in Japan is needed to consider evidence-based health policies to prevent essential hypertension for Japanese women. In this study, we aimed to clarify the association between GH (blood pressure ≥140/90 mmHg without proteinuria after the 20th week of gestation) in the first pregnancy and future hypertension in middle-aged or older women.

## 2. Materials and Methods

### 2.1. Study Design and Subjects

This case-control study was performed from April 2018 to March 2020. The case group was composed of women recruited from three settings: (1) outpatients to the Department of Nephrology and Hypertension at Dokkyo Medical University Hospital; (2) outpatients to the Department of Cardiovascular Medicine and Nephrology, Dokkyo Medical University Nikko Medical Center; and (3) residents who completed an annual health check-up in the town of Mibu, Tochigi prefecture (approximately 100 km northeast of Tokyo). According to the National Health and Nutrition Survey 2016, the difference between BMI, salt intake, and vegetable intake between Tochigi and Japan was small, as described in the discussion [20]. A total of 62 hypertensive cases (24 hypertensive outpatients and 38 hypertensive residents) aged 44–85 years who completed a questionnaire survey and kept their Maternal and Child Health Handbook (MCH) of the first pregnancy were included in the case group.

Of the residents who completed the annual health check-up in Mibu, a total of 75 women aged 40–73 years without hypertension and with MCH records of their first pregnancy were selected in the control group.

### 2.2. Questionnaire

The questionnaire had two parts. The first part was the self-administered questionnaire about the current status of the subjects. The questionnaire included the following items: current age, height and weight, birth weight of the subject herself, medical history of the subject (hypertension, diabetes, dyslipidemia, renal disorder, etc.), and birth history (number of pregnancy, number of births, height and weight of subject before the first pregnancy, complications of HDP and gestational diabetes mellitus during the first pregnancy, weight change of subject after the first pregnancy). The second part asked about data at the first pregnancy that were obtained from MCH at the first pregnancy. The items were as follows: observation results at each prenatal checkup (fundal height, abdominal circumference, body-weight blood pressure, edema, proteinuria, urinary sugar), and outcome of delivery (gestational weight at delivery, mode of delivery, birth weight of the child, etc.).

GH and PE were determined by obstetrics and gynecology physicians using the answers to the self-administered questionnaires and MCH data. Data related to gestational week, systolic blood pressure, diastolic blood pressure, and proteinuria at each visit during pregnancy were obtained from the prenatal checkup record of MCH.

### 2.3. Definitions

Hypertension was defined according to the criteria (systolic blood pressure ≥ 140 mmHg and/or diastolic blood pressure ≥ 90 mmHg) described in a report of the American College of Cardiology/American Heart Association Task Force on Clinical Practice Guidelines [1] or taking antihypertensive medications.

According to the criteria of the American College of Obstetricians and Gynecologists in 2013 [9], HDP was defined as hypertension in pregnancy. GH was defined as the presence of hypertension (blood pressure ≥140/90 mmHg) without proteinuria after the 20th week of gestation. PE was defined as the presence of hypertension (blood pressure ≥140/90 mmHg) and proteinuria (proteinuria ≥ 300 mg/day) after the 20th week of gestation. However, in this study, PE was defined as the continued observation of hypertension and proteinuria (+ or more). In addition, early-onset GH was diagnosed as the onset of hypertension after the 20th week of gestation and before the 34th week of gestation. Late-onset GH was defined as the onset of hypertension after the 34th week of gestation [21].

### 2.4. Statistical Analysis

To analyze differences in characteristics between the case and control groups, statistical analyses were performed using either the Student’s t-test or the Mann–Whitney U-test. Differences in binominal items were assessed using Fisher’s exact test. Logistic-regression analysis was performed in univariate and multivariable analyses. To avoid overfitting in multivariable analysis, covariables were entered using potential confounding factors for essential hypertension, such as age and body-mass index (BMI) at recruitment (Model 1). In addition, Model 2 was adjusted for covariates, viz., BMI and age at recruitment, prepregnancy BMI, and age at the first delivery. All statistical analyses were performed using IBM SPSS version 25 (IBM Japan, Ltd., Tokyo, Japan).

### 2.5. Ethics

All participants provided written informed consent, and the study was approved by the Institutional Review Board of Dokkyo Medical University (Daigaku 29011), Dokkyo Medical University Hospital (R-17-13J), and Dokkyo Medical University Nikko Medical Center (Nikko 21005).

## 3. Results

The characteristics of the 137 subjects are shown in Table 1. Mean (standard deviation (SD)) is presented for continuous variables. The case group was significantly older than the control group at the time of recruitment (63.1 (8.4) vs. 57.7 (9.4), *p* < 0.001). The BMI at recruitment was significantly higher in the case group than in the control group (24.1 (5.2) vs. 21.6 (2.9), *p* < 0.001). The history of diabetes mellitus was significantly higher in the case group than in the control group (11.3% vs. 0%). The prepregnancy BMI was also significantly higher in the case group than in the control group (21.5 (3.3) vs. 20.4 (1.8), *p* = 0.015). No significant differences in the history of dyslipidemia, history of renal disorders, age at the first delivery, mothers’ weight at delivery, weight changes after the first pregnancy, mode of delivery, gestational age (weeks) at delivery, and child’s birth weight between the two groups were recorded.

Of the 137 subjects, 26 (19.0%) had HDP, which consisted of 17 GH, 1 PE, and 8 unclassifiable HDP. The ORs of hypertension according to HDP subtypes are shown in Table 2. Compared with normal pregnancy, the crude OR for GH was significantly high (OR 7.7, 95% CI 2.1–28.3). Even after adjusting for age and BMI at recruitment, the OR for GH remained significant (OR 4.8, 95% CI 1.2–18.9). The OR for GH was 4.2 (95% CI 1.0–17.5) when age and body-mass index at recruitment, age at delivery, and prepregnancy body-mass index were adjusted.

Crude ORs in the early and late onset were significantly associated with future hypertension; however, the age- and BMI-at-recruitment-adjusted ORs of both types were not significant (Table 3).

## 4. Discussion

This study showed that GH during the first pregnancy was associated with future hypertension among Japanese women. The OR remained significant even after adjustment for age and BMI at recruitment. The OR narrowly failed to remain significant after adjustment for age and BMI at recruitment, prepregnancy BMI, and age at the first delivery. If a significant relationship between GH and hypertension could be found after adjusting these GH risk factors with age and BMI at recruitment, it would mean GH might be an independent risk factor. The ORs remained significant after adjustment for age and BMI at recruitment, and age at delivery, and after adjustment for age and BMI at recruitment, and prepregnancy BMI (data not shown). In this study, the significant disappearance might have been because of the small sample size.

Although differences existed in the diagnosis of GH in past pregnancies as described in Japanese studies [18,19], the results of the present study were consistent with these previous studies. Other studies [2,3,4,5,6,7,8] could also support our results; thus, GH during the first pregnancy is likely to be an independent risk factor for future hypertension.

This study has some particular points. In this study, data of blood pressure and proteinuria during pregnancy were obtained from the MCH. In Japan, MCH has been used in children and material health check-up since 1947 [22]. For MCH, blood pressure and proteinuria were recorded by obstetricians. Therefore, the recall bias could be quite low, and results could be reliable. In multivariable-logistic-regression analysis, strong and certain association of GH with future hypertension was found after adjustment for age at recruitment, age at the first delivery, prepregnancy BMI, and BMI at recruitment. Furthermore, we divided the cases into two by period of onset because the early-onset type, which is characterized with an inadequate trophoblastic invasion of the uterine spiral arteries and the narrowing of spiral arterioles in early pregnancy [16], did not yield significant results because of few events, although crude analysis indicated positive associations with the risk of hypertension in both types. Therefore, we did not find any differences between the timing of the onset of GH.

Obesity is a known risk factor of hypertension [23]. In this study, significant association of GH with future hypertension remained even after adjustment of BMI at recruitment. This finding may suggest that GH could be a risk factor independent of BMI. Several lifestyles, such as high sodium intake and lack of physical activity, are also known major risk factors of hypertension [24,25]. Further studies with a larger sample size are warranted to clarify the pathogenesis of the association of GH with hypertension.

In this study, GH and PE were discussed because of their differences in pathogenesis and condition. Over the decades, GH was assumed to cause an overload beyond a threshold of a “stress test” in pregnancy on the basis of some genetic factors of the mothers [26]. Conversely, several recent studies of the M235T variant of angiotensinogen gene (*AGT*) found that T235 of *AGT* may cause the onset of PE by strongly linking with the A(-6) of another single-nucleotide polymorphism (SNP) of the promotor region G(-6)A that could cause placental hypoplasia [11,14,15,16,17]. In short, GH, which does not tend to be based on these SNPs of *AGT*, might be strongly associated with future hypertension. In some case-control studies in the Japanese population, the T235 of *AGT* was found to be associated with PE rather than GH, and to be an independent risk factor among other risk factors for hypertension, such as obesity or a family history of hypertension [17,27,28].

The mechanisms of association between GH and future hypertension remain unknown. According to recent molecular epidemiological studies, in addition to past observational studies, many possible hypotheses were discussed about the pathogenesis of HDP, for example, placental dysfunction is caused by hypoplasia of the spiral artery related to the M235T variant of *AGT,* and the SNP of promoter region G(-6)A of *AGT* [11,12,13,14,15,16,17]. However, nowadays, HDP is considered a syndrome of several types following the interactions of several genetic and environmental factors [27]. Essential hypertension could be caused by complex pathogeneses that are related to some genetic and environmental factors. Recent meta-analyses of genetic factors suggested that β3-adrenergic receptor gene Trp64Arg (T64A) polymorphism may be associated with an increased risk for essential hypertension by reducing cellular-signal transduction from repair that prevents adipose-tissue heat production and decomposition [29], and alpha epithelial sodium channel gene (*alpha-ENaC*) T663A polymorphism may also be associated with essential hypertension through pathogenesis concerning sodium reabsorption [30]. Polymorphism related to sodium sensitivity might be a common risk factor for both GH and essential hypertension. Further studies to elucidate differences in gene–environment interactions between GH and future hypertension are warranted.

This study had some limitations. First, we could not examine differences in the influence between GH and PE because of the small sample size, so the focus of this study was the association between GH and essential hypertension in middle-aged or older women. Second, we could not obtain reliable information about the family history of hypertension given the possibility of recall bias when obtaining data from middle-aged to older cases and controls. Finally, the generalizability of our study results would not be perfect because the study was conducted in a prefecture. However, the findings of our study may apply to Japan because differences in BMI, salt intake, and vegetable intake between Tochigi and Japan were small. Concretely, according to the National Health and Nutrition Survey 2016, the mean body-mass index was 23.9 kg/m^2^ for men in Tochigi versus 23.8 kg/m^2^ for men in Japan, and 22.8 kg/m^2^ for women in Tochigi versus 22.6 kg/m^2^ for women in Japan. Mean salt intake was 10.6 g/day for men in Tochigi versus 10.8 g/day for men in Japan, and 9.0 g/day for women in Tochigi versus 9.2 g/day for women in Japan. Mean vegetable intake was 279.5 g/day for men in Tochigi versus 284.2 g/day for men in Japan, and 277.2 g/day for women in Tochigi versus 270 g/day for women in Japan [20].

## 5. Conclusions

From the viewpoint of cardiovascular-disease prevention, identification of high-risk women for future hypertension during pregnancy could be of great value to decrease the incidence of cardiovascular diseases. The results of this study might suggest a possibility of GH to identify high-risk women for future hypertension. Obstetricians could encourage high-risk postpartum women to adopt a healthier lifestyle, such as by reducing salt intake and performing moderate exercises, measuring blood pressure daily, and having at least annual health checkups to prevent future essential hypertension. It is necessary to discuss this topic in the future. In conclusion, GH might be an independent risk factor of future hypertension in middle-aged-to-older Japanese women. Future studies are warranted to elucidate the detailed mechanisms, and to suggest a novel health intervention to women with a history of HDP.

## Figures and Tables

**Table 1:** Characteristics of participants (N = 137).

	Cases	Controls	*p*-Values^1^
	(*N* = 62)	(*N* = 75)	
Age at recruitment, mean (SD), years	63.1 (8.4)	57.7 (9.4)	<0.001
Body-mass index at recruitment, mean (SD), kg/m^2^	24.1 (5.2)	21.6 (2.9)	<0.001
History of diabetes mellitus			0.003
No	55 (88.7)	75 (100.0)	
Yes	7 (11.3)	0 (0.0)	
History of dyslipidemia			0.648
No	45 (72.6)	57 (76.0)	
Yes	17 (27.4)	18 (24.0)	
History of renal disorders			0.090
No	59 (95.2)	75 (100.0)	
Yes	3 (4.8)	0 (0.0)	
Birth weight of participants themselves, g, n (%)			0.296
< 2500	4 (7.8)	2 (2.7)	
2500–2999	27 (52.9)	28 (37.3)	
3000–3499	18 (35.3)	26 (34.7)	
3500–4000	2 (3.9)	7 (9.3)	
≥ 4000	0 (0.0)	0 (0.0)	
History of hypertensive disorder of pregnancy, n (%)			<0.001
No	42 (67.7)	69 (93.2)	
Yes	20 (32.3)	6 (8.0)	
Weight change after first delivery versus prepregnancy weight, n (%)			0.476
Stable	25 (40.3)	34 (45.3)	
Increase	30 (48.4)	29 (38.7)	
Decrease	7 (11.3)	12 (16.0)	
Prepregnancy body-mass index, mean (SD), kg/m^2^	21.5 (3.3)	20.4 (1.8)	0.015
Age at first delivery, mean (SD), years	26.9 (3.9)	28.2 (4.2)	0.075
Mode of delivery ^2^, n (%)			0.801
Cesarean section	5 (8.3)	7 (9.6)	
Other	55 (91.7)	66 (90.4)	
Gestational week at delivery, mean (SD), week	39.6 (1.6)	39.3 (1.8)	0.458
Birth weight of the child ^2^, g, n (%)			0.887
< 2500	3 (4.9)	5 (6.7)	
2500–2999	20 (32.8)	30 (40.0)	
3000–3499	26 (42.6)	28 (37.3)	
3500–4000	11 (18.0)	11 (14.7)	
≥ 4000	1 (1.6)	1 (1.3)	

Values presented as mean (standard deviation) or number (percentage). ^1^ Student’s t-test, chi-squared test, or Fisher’s exact test. ^2^ Some data were excluded due to missing values.

**Table 2:** Risks of hypertension according to HDP subtypes.

	No. of Cases/Controls	Crude		*p*-Value	Model 1 ^1^		*p*-Value	Model 2 ^2^		*p*-Value
		OR	(95% CI)		OR	(95% CI)		OR	(95% CI)	
HDP										
No	42/69	1.0			1.0			1.0		
Yes										
GH	14/3	7.7	(2.1–28.3)	0.002	4.8	(1.2–18.9)	0.024	4.2	(1.0–17.5)	0.051
PE	0/1									
Another type	6/2	4.9	(1.0–25.6	0.058	3.7	(0.7–19.9)	0.132	3.2	(0.5–20.1)	0.220

CI, confidence interval; GH, gestational hypertension; HDP, hypertensive disorders of pregnancy; OR, odds ratio; PE, pre-eclampsia. ^1^Adjusted for age and body-mass index at recruitment. ^2^Adjusted for age and body-mass index at recruitment, age at delivery, and prepregnancy body-mass index.

**Table 3:** Risks of hypertension according to timing of GH onset.

	No. of Cases/Controls	Crude		*p*-Value	Adjusted ^1^		*p*-Value
		OR	(95% CI)		OR	(95% CI)	
non-GH	42/69	1.0			1.0		
GH							
Early onset	7/2	5.8	(1.1–29.0)	0.034	3.6	(0.7–19.3)	0.130
Late onset	7/1	11.5	(1.4–96.8)	0.025	7.1	(0.8–63.7)	0.080

CI, confidence interval; GH, gestational hypertension; OR, odds ratio. Early onset was diagnosed before 34^th^ week of gestation. ^1^Adjusted for age and body-mass index at recruitment.
